# Colorimetric quantification of sucrose in presence of thermo-sensitive polymers present in aqueous two-phase systems

**DOI:** 10.1016/j.mex.2014.09.006

**Published:** 2014-10-08

**Authors:** Subbarayalu Ramalakshmi, Chien Wei Ooi, Arbakariya B. Ariff, Ramakrishnan Nagasundara Ramanan

**Affiliations:** a Chemical Engineering Discipline, School of Engineering, Monash University Malaysia, Jalan Lagoon Selatan, 47500 Bandar Sunway, Selangor, Malaysia; b Department of Bioprocess Technology, Faculty of Biotechnology and Biomolecular Sciences, Universiti Putra Malaysia, 43400 UPM Serdang, Selangor, Malaysia; c Advanced Engineering Platform, School of Engineering, Monash University Malaysia, Jalan Lagoon Selatan, 47500 Bandar Sunway, Selangor, Malaysia

**Keywords:** Aqueous two-phase system, Biomolecule purification, Colorimetric methods, Liquid–liquid equilibrium, Triton

## Abstract

The use of biodegradable material such as simple carbohydrates and recyclable material such as thermo-sensitive polymers is in need to develop a sustainable aqueous two-phase system (ATPS) for the purification of biomolecules. Accurate determination of sucrose concentration is important in liquid–liquid equilibrium (LLE) study of carbohydrate-based ATPS. The well-established phenol–sulfuric acid method has been widely employed in the measurement of carbohydrate concentration. However, the presence of thermo-sensitive polymers, which has a lower critical solution temperature (LCST) below room temperature, in carbohydrate samples could hamper the precision of spectrophotometric analysis due to the formation of two phases or cloudiness in the sample. Thus, the following modifications were made in an attempt to eliminate the interference occurred during conventional phenol–sulfuric acid assay.

• The modified assay for sucrose quantification was performed at an ice-cold temperature throughout the reaction in order to avoid the interference from thermo-sensitive polymers.
• This method required a sample volume of 3 μL and hence the volume of other reagents employed was also considerably reduced.
• The absorbance was measured at 520 nm which allowed a longer linearity range (0.05–7.5%, w/v).

## Graphical abstract

**Figure:**
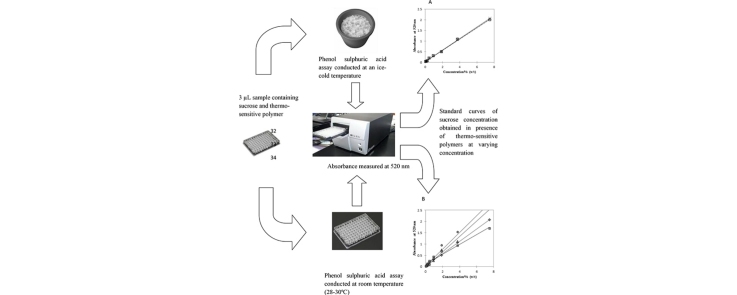

## Materials

• EO50PO50 (Sigma, Cat. No. 438189)
• Phenol (Scharlau, Cat. No. FE04821000)
• Sucrose (Sigma, Cat. No. S5106)
• Sulfuric acid (Merck, Cat. No. 1.00731.1000)
• Triton X-100 (Sigma, Cat. No. T9284)
• Triton X-114 (Sigma, Cat. No. X144)

Ultra-pure water was used throughout the experiment. Sucrose standards were prepared by mixing equal amounts of sucrose (final concentration of 0.05–7.5%, w/v) and ultra-pure water. The mixtures were placed at room temperature (28–30 °C). Sample mixtures were prepared by mixing equal amounts of sucrose and thermo-sensitive polymers with a final concentration ranging from 0.05 to 7.5% (w/v) and 0.05 to 5% (w/v), respectively. The mixtures were then placed in a chiller at 4 °C for 1 h to prevent the phase formation in the sample solution. To study the interference contributed by three thermo-sensitive substances in sucrose quantification, samples were prepared by mixing equal volumes of sucrose (final concentration of 0.05–7.5%, w/v) and 0.5% (w/v) of each thermo-sensitive polymers. The mixtures were placed at room temperature for 1 h before the assay was carried out.

## Method details

1. The modified phenol–sulfuric acid assay was performed in a 96-well microplate, maintained at an ice-cold temperature.
2. A 75 μL of concentrated sulfuric acid (96–98%) was added to 3 μL of sample mixture.
3. This was followed by the addition of 15 μl of 5% (w/v) phenol.
4. The mixture was shaken for about 5 min and the absorbance was measured at 520 nm using a microplate reader (Sunrise, Tecan).
5. Assay for the reference samples was conducted at room temperature instead of in an ice bath and the absorbance was measured at 520 nm.

**Fig. 1:**
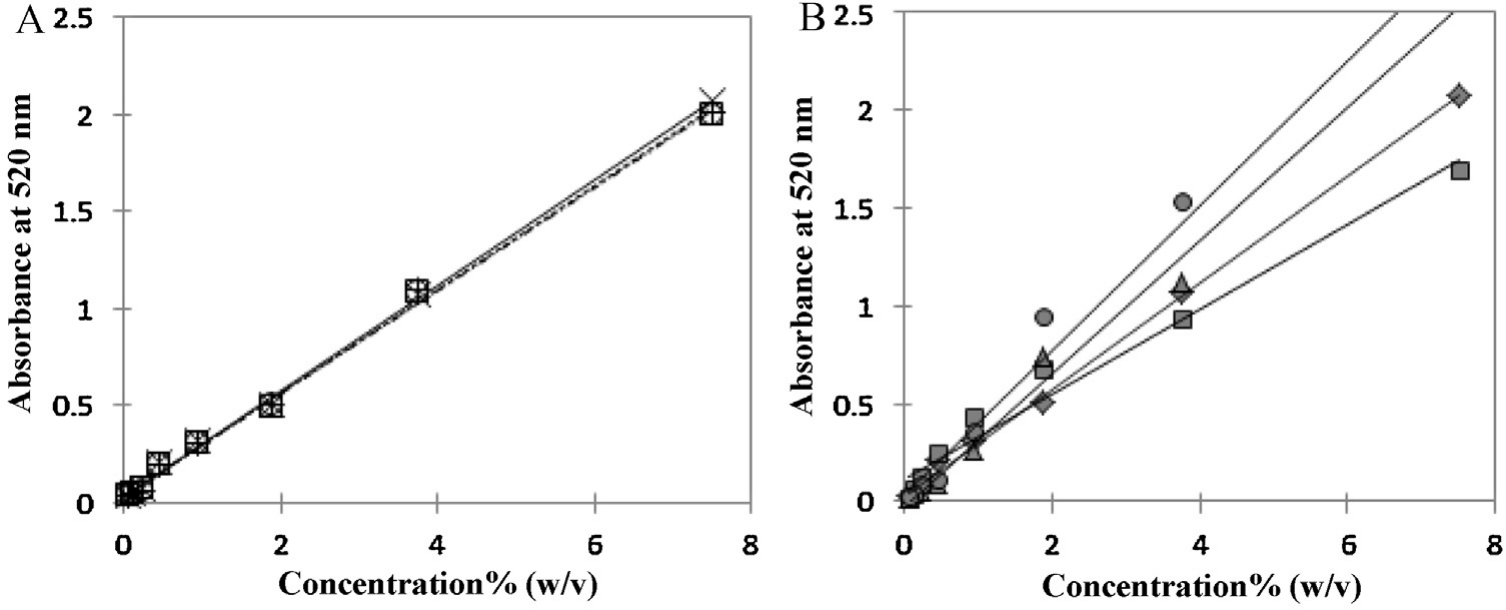
Standard curves of phenol–sulfuric acid method conducted at an ice-cold temperature (A) and room temperature (B). (A) -×-: sucrose; ⋯+⋯: sucrose + Triton X-114 (0.05%, w/v); ⋯□⋯: sucrose + Triton X-114 (0.5%, w/v); ⋯○⋯: sucrose + Triton X-114 (5%, w/v); (B) -♦-: sucrose; -■-: sucrose + Triton X-114 (0.5%, w/v); -▴- sucrose + Triton X-100 (0.5%, w/v); -●-: sucrose + EO50PO50 (0.5%, w/v)

In this assay, initial temperature of the sample in the 96-well microplate was 8 °C which rose to 32 °C with the addition of sulfuric acid and later reduced to 23 °C after the condensation of phenol with furfural derivatives. In such range of temperature, the sample mixture did not form any cloudiness or two-phases. Standard curves obtained from the assay conducted at an ice-cold temperature and room temperature are shown in Fig. 1(A) and (B), respectively. From Fig. 1(A) and (B), it could be seen that the assays carried out at an ice-cold temperature exhibited a very similar standard curve whereas a significant difference among the slopes of standard curves was observed for the assays conducted at room temperature. Standard curves of solution containing sucrose + Triton X-100 and sucrose + EO50PO50 mixtures at an ice-cold temperature are attached as supplementary figures (Figs. 2 and 3). The values of co-efficient of determination (*R*^2^), slope and standard deviation (SD) of phenol–sulfuric acid method conducted at an ice-cold temperature and room temperature are tabulated in Table 1(A) and (B), respectively. *R*^2^ values represent the best fitting of data points in a line and the values of *R*^2^ in Table 1(A) and (B) are greater than 0.97. SD denotes the measure of deviation of values of slope of respective samples from the average value of slope of standard sucrose (mean). The deviation of slope values in Table 1(B) is higher than in Table 1(A). Therefore, we infer that there is significant interference when the assay was conducted at room temperature.

In conclusion, the low temperature phenol–sulfuric acid method offers accurate measurement of sucrose concentration in presence of thermo-sensitive polymers. Moreover, in our work, all steps of the assay were performed at a constant ice-cold temperature and hence the time consumed for the whole assay is less when compared to all existing methods [1–3]. The values of SD (less than 0.002) and *R*^2^ (0.9982) show the accuracy and linearity of the revised method, respectively. Hence, this low temperature phenol–sulfuric acid method can be extended to the measurement of concentration of other types of carbohydrates in solutions containing thermo-sensitive polymers.

## Additional background information

Thermo-sensitive polymers such as Triton X-114, Triton X-100 and EO50PO50 [a random copolymer of 50% ethylene oxide (EO) and 50% propylene oxide (PO)] are widely employed as phase-forming reagents in ATPS because the polymers separate into two phases when the solution is heated above the lower critical solution temperature (LCST). LCSTs of Triton X-114, Triton X-100 and EO50PO50 are 28 °C, 67 °C and 55 °C respectively [4–6]. In carbohydrate-based ATPSs, sugars are used as one of the phase-forming components [7]. Measurement of concentration of the phase-forming components is essential for tie-line construction in ATPS study. Concentration of sugars in the phases can be determined with the help of chromatographic [8,9], physical [10,11] and colorimetric methods [1–3,12]. Among all methods of carbohydrate analysis, colorimetric methods have the advantages such as less time consumption, inexpensive, high throughput and easy workflow. Phenol–sulfuric acid method is one of the colorimetric methods [1–3] best suited when the sample mixture has sugars along with polymers [13], proteins [14] or salts [15]. Moreover, in phenol–sulfuric acid method, the color formation is stable for a longer period resulting in more accuracy when compared to other colorimetric methods [3].

The process temperature employed in all existing phenol–sulfuric acid methods [1–3] was not favorable for the measurement of sucrose concentration in presence of thermo-sensitive polymers. This is due to the formation of cloudiness and hence these methods have less tolerance to interference from thermo-sensitive materials in sucrose concentration measurement. Therefore in our work, the temperature was maintained below the LCST of thermo-sensitive polymers by carrying out the assay in a 96-well microplate which was maintained at an ice-cold temperature.

## Figures and Tables

**Table 1:** Linearity of standards for phenol–sulfuric acid method at an ice-cold temperature (A) and room temperature (B).^a^

Sample mixtures	Slope	*R*^2^	SD of the slope
**A**			
Sucrose standard (0.05–7.5%, w/v)	0.1367	0.9982	–
Sucrose + Triton X-114 (5%, w/v)	0.1336	0.9975	0.002
Sucrose + Triton X-114 (0.5%, w/v)	0.1327	0.9974	0.002
Sucrose + Triton X-114 (0.05%, w/v)	0.1327	0.9974	0.002
Sucrose + Triton X-100 (5%, w/v)	0.1385	0.9992	0.001
Sucrose + Triton X-100 (0.5%, w/v)	0.1366	0.9982	0
Sucrose + Triton X-100 (0.05%, w/v)	0.1378	0.9974	0.0007
Sucrose + EO50PO50 (5%, w/v)	0.1356	0.9984	0.0008
Sucrose + EO50PO50 (0.5%, w/v)	0.1353	0.9981	0.0009
Sucrose + EO50PO50 (0.05%, w/v)	0.1344	0.9981	0.002

**B**			
Sucrose + Triton X-114 (0.5%, w/v)	0.1082	0.9746	0.02
Sucrose + Triton X-100 (0.5%, w/v)	0.1703	0.9931	0.03
Sucrose + EO50PO50 (0.5%, w/v)	0.1855	0.9874	0.02

a Low temperature phenol–sulfuric acid method was conducted at an ice-cooled temperature and conventional phenol–sulfuric acid method was conducted at room temperature.
