# Supplementary material for: Colorimetric quantification of sucrose in presence of thermo-sensitive polymers present in aqueous two-phase systems
Source: MethodsX. 2014 Oct 8;1:229–32. doi: 10.1016/j.mex.2014.09.006 (PMC4472962; doi:10.1016/j.mex.2014.09.006)
Supplement: Supplementary file 1 [file mmc1.doc]

**Supplementary figures**

**Fig. 2 caption**

Standard curves of low temperature phenol-sulfuric acid assay for sucrose+Triton X-100

**Fig. 2. Legends**

-×-: sucrose; ˗ ˗+˗ ˗: sucrose+Triton X-100 (0.05%, w/v); …□…: sucrose+Triton X-100 (0.5%, w/v); - -○- -: sucrose+Triton X-100 (5%, w/v)

**Fig. 3 caption**

Standard curves of low temperature phenol-sulfuric acid assay for sucrose+EO50PO50

**Fig. 3. Legends**

-×**-**: sucrose; ˗ ˗+˗ ˗: sucrose+EO50PO50 (0.05%, w/v); …□…: sucrose+EO50PO50 (0.5%, w/v); - -○- -: sucrose+ EO50PO50 (5%, w/v)

**Fig. 2**

**Fig. 3**
